# Single-pulse electrical stimulation artifact removal using the novel matching pursuit-based artifact reconstruction and removal method (MPARRM)

**DOI:** 10.1088/1741-2552/ad1385

**Published:** 2023-12-27

**Authors:** Tao Xie, Thomas J Foutz, Markus Adamek, James R Swift, Cory S Inman, Joseph R Manns, Eric C Leuthardt, Jon T Willie, Peter Brunner

**Affiliations:** 1 Department of Neurosurgery, Washington University School of Medicine, St. Louis, MO, United States of America; 2 National Center for Adaptive Neurotechnologies, St. Louis, MO, United States of America; 3 Department of Neurology, Washington University School of Medicine, St. Louis, MO, United States of America; 4 Department of Neuroscience, Washington University School of Medicine, St. Louis, MO, United States of America; 5 Department of Psychology, University of Utah, Salt Lake City, UT, United States of America; 6 Department of Psychology, Emory University, Atlanta, GA, United States of America

**Keywords:** Single-pulse electrical stimulation, matching pursuit, broadband gamma, early response, CCEP, stimulation artifact removal, CCSR

## Abstract

*Objective.* Single-pulse electrical stimulation (SPES) has been widely used to probe effective connectivity. However, analysis of the neural response is often confounded by stimulation artifacts. We developed a novel matching pursuit-based artifact reconstruction and removal method (MPARRM) capable of removing artifacts from stimulation-artifact-affected electrophysiological signals. *Approach.* To validate MPARRM across a wide range of potential stimulation artifact types, we performed a bench-top experiment in which we suspended electrodes in a saline solution to generate 110 types of real-world stimulation artifacts. We then added the generated stimulation artifacts to ground truth signals (stereoelectroencephalography signals from nine human subjects recorded during a receptive speech task), applied MPARRM to the combined signal, and compared the resultant denoised signal with the ground truth signal. We further applied MPARRM to artifact-affected neural signals recorded from the hippocampus while performing SPES on the ipsilateral basolateral amygdala in nine human subjects. *Main results.* MPARRM could remove stimulation artifacts without introducing spectral leakage or temporal spread. It accommodated variable stimulation parameters and recovered the early response to SPES within a wide range of frequency bands. Specifically, in the early response period (5–10 ms following stimulation onset), we found that the broadband gamma power (70–170 Hz) of the denoised signal was highly correlated with the ground truth signal ($R = 0.98\pm0.02$, Pearson), and the broadband gamma activity of the denoised signal faithfully revealed the responses to the auditory stimuli within the ground truth signal with $94\%\pm1.47\%$ sensitivity and $99\%\pm1.01\%$ specificity. We further found that MPARRM could reveal the expected temporal progression of broadband gamma activity along the anterior-posterior axis of the hippocampus in response to the ipsilateral amygdala stimulation. *Significance.* MPARRM could faithfully remove SPES artifacts without confounding the electrophysiological signal components, especially during the early-response period. This method can facilitate the understanding of the neural response mechanisms of SPES.

## 1. Introduction

The neural responses to single-pulse electrical stimulation (SPES) can provide evidence for direct and indirect structural and functional connectivity (Matsumoto *et al* 2017, Crowther *et al* 2019, Parmigiani *et al* 2022, Sawada *et al* 2022). These responses include cortico-cortical evoked potentials (CCEPs) and cortico-cortical spectral responses (CCSRs). CCEPs consist of phase-locked responses revealed by averaging signals in the temporal domain (Matsumoto *et al* 2004, Matsumoto *et al* 2017, Keller *et al* 2014), while CCSRs are dominated by non-phase-locked neural responses obtained by averaging activity in canonical frequency bands in the spectral domain (Usami *et al* 2019a, 2019b, Sugiura *et al* 2020). Recently, there has been a significant surge of interest in exploring CCSRs to enable more precise interpretation of SPES-responses (Usami *et al* 2015, 2019a, Crowther *et al* 2019). Specifically, neural activity within the broadband gamma band (70–170 Hz) is of particular neuroscientific interest. Studies have shown that broadband gamma activity is correlated with population-level activity that represents underlying local multi-unit spiking activity (Nir *et al* 2007, Ray *et al* 2008a, Manning *et al* 2009, Miller *et al* 2014). However, spectral response analysis is often confounded by stimulation artifacts that can masquerade as physiological responses due to spectral leakage or temporal spread.

SPES is typically delivered as a 0.1–1 ms-long constant-current square-wave pulse at a rate of 1 Hz or lower. Like any other direct electrical stimulation, SPES creates large stimulation artifacts at the stimulation onset (Matsumoto *et al* 2017). These artifacts are characterized by a sharp morphology and broad spectral power increase akin to a Dirac function, followed by a slower capacitive discharge, which can masquerade as a physiological response to electrical stimulation. Acquiring these signals using biosignal amplifiers and analog-to-digital converters with inherent filtering further spreads this stimulation artifact in time and frequency. SPES studies generally exclude the early response period (5–20 ms post-stimulation) to prevent stimulation artifacts from contaminating their signal analysis (Trebaul *et al* 2016, Crowther *et al* 2019, Toth *et al* 2020). However, early responses might reflect monosynaptic or oligosynaptic connectivity (Logothetis *et al* 2010, Keller *et al* 2014, Toth *et al* 2020, Kudela and Anderson 2021), and could potentially be used to determine effective functional connectivity within local neural circuits such as the amygdala-hippocampus circuit (Inman *et al* 2018), temporal lobe (Novitskaya *et al* 2020), thalamic motor nuclei (Toth *et al* 2020), subthalamic nucleus or globus pallidus internus (Sinclair *et al* 2019, Schmidt *et al* 2020, Connolly *et al* 2022, Dale *et al* 2022). Thus, there is an imperative need to develop an effective stimulation artifact removal methodology to permit the analysis of the physiological components within early responses to SPES.

Existing stimulation artifact removal methods are primarily based on interpolation, template subtraction, and model decomposition. Interpolation methods substitute the artifact-affected signal with linear interpolation (Voigt *et al* 2018), curve fitting (Wagenaar and Potter 2002, David *et al* 2013), or linear merging of surrounding signals (Crowther *et al* 2019). Template subtraction methods subtract an approximation of the stimulation artifact (i.e. the template) from each individual stimulation trial. Artifact templates are typically determined by averaging artifacts across trials (Wichmann 2000, Hashimoto *et al* 2002, Erez *et al* 2010, Sun and Hinrichs 2016, Hammer *et al* 2022), or by using machine learning (Alagapan *et al* 2019), biophysical models (Trebaul *et al* 2016) and dictionary learning (Caldwell *et al* 2020). Model decomposition methods will decompose the artifact-affected neural signal into the artifact and denoised signal by applying an independent component analysis (Gilley *et al* 2006, Lu *et al* 2012, Rogasch *et al* 2014), principal component analysis (ter Braack *et al* 2013, O’Shea and Shenoy 2018), or Gaussian processes (Mena *et al* 2017).

While previously published stimulation-artifact removal methods have been applied with variable success to distinguish CCEPs, there is limited evidence for their validity in preserving early spectral responses (especially in the broadband gamma range) that occur within the first 20 ms, limiting their utility in the precise physiological interpretation of SPES (Crowther *et al* 2019). Specifically, because of the close temporal proximity between the stimulation artifact and early neural responses, any residual artifact could result in spurious early spectral responses in the broadband gamma range. This is further exacerbated by the uncertainty principle (Folland and Sitaram 1997), which temporally spreads any residual stimulation artifact in the course of filtering the signal.

**Figure 1.**
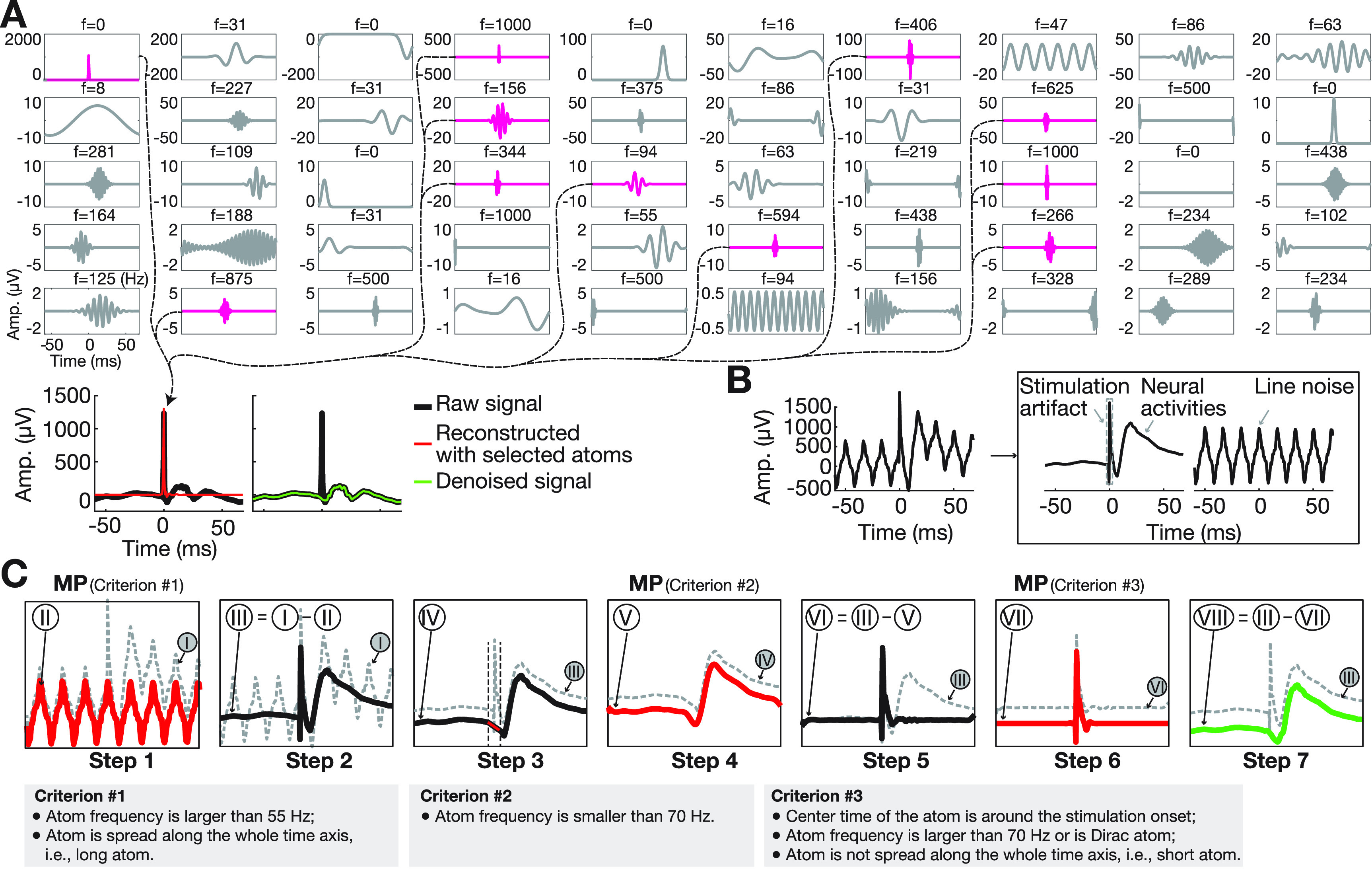
Schematic overview of matching pursuit-based artifact reconstruction and removal method (MPARRM). (A) The matching pursuit (MP) algorithm iteratively decomposes a signal into a linear combination of basis functions (i.e. atoms). In a simplified denoising procedure, the raw signal (black) containing a strong individual stimulation artifact around time 0 is decomposed into 50 atoms using the MP algorithm (Chandran KS *et al* 2016). Selecting atoms representing stimulation artifacts (magenta) and reconstructed individual stimulation artifact (red). Removing the reconstructed stimulation artifact from the raw signal yields a denoised signal (green). (B) Brain signals recorded during electrical stimulation are comprised of three major components, including electrical line noise, sharp stimulation artifact, and neural activity (i.e. rhythmic and transient electrophysiological signals). (C) Description of MPARRM. Dashed grey trace (Roman numeral-I) represents the raw signal. The seven steps within MPARRM yield the final denoised signal represented by the green trace (Roman numeral-VIII). Line noise and the stimulation artifact have been removed, while neural activity has been preserved.

To address these limitations and to faithfully extract early spectral stimulation responses, we developed a novel matching pursuit (MP)-based artifact reconstruction and removal method (MPARRM, figure 1). The MP algorithm has the distinct advantage of ‘separating’ the stimulation artifact, which is characterized by sharp morphology, from the electrophysiological signals without creating spurious spectral responses, something that is difficult to accomplish with conventional methods such as IIR/FIR filters, short-time Fourier transform, or wavelet transform (Chandran KS *et al* 2016). MPARRM accomplishes this by first reconstructing each stimulation artifact based on the general characteristics of the signal in the temporal and spectral domain, followed by removing each individual reconstructed stimulation artifact from the signal in the temporal domain.

We first tested MPARRM in a bench-top study to verify the ability to remove stimulation artifacts. We then applied MPARRM in an in-vivo study on neural signals recorded from the human amygdala-hippocampus circuit during basolateral amygdala stimulation. The results of our study showed that MPARRM could faithfully preserve early spectral responses to SPES, and facilitate studies that advance our understanding of effective functional connectivity within local neural circuits.

## 2. Materials and methods

### 2.1. Overview of MPARRM

The ‘uncertainty principle’ (Folland and Sitaram 1997) imposes an upper limit to the accuracy in the representation of signals in the spectral domain. In practice, this principle creates the necessity for a temporal observation window to estimate the spectral decomposition of a signal, thereby imposing a constraint on the accuracy with which the onset of a signal can be determined. For example, a short rectangular pulse (similar to a digital Dirac impulse), with a well-defined onset in the temporal domain, becomes a sinc-function in the spectral domain, with a less-well-defined onset. This Dirac-to-sinc issue becomes problematic when analyzing CCSRs, as any residual artifact (supplementary figure 1) could result in a spurious spectral response around the stimulation onset (including the early response period). For example, when using the conventional interpolation method (Crowther *et al* 2019), we observe a spurious spectral response around stimulation onset (figure 3(B)). Thus, simply ‘replacing’ the artifact around the stimulation onset with an interpolated signal leaves a residual artifact. MPARRM addressed this issue by ‘separating’ the stimulation artifact from the artifact-affected signal while preserving the spectral responses around stimulation onset.

**Figure 2.**
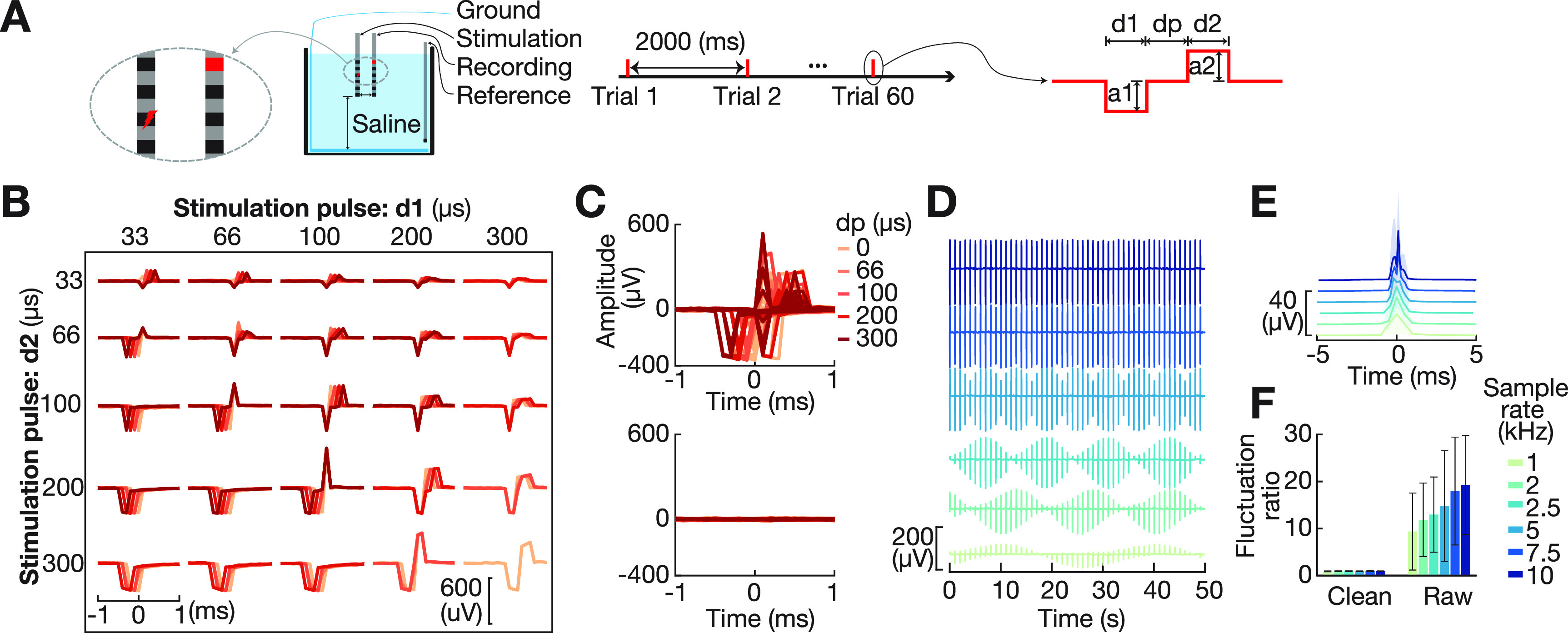
Single-pulse electrical stimulation (SPES) artifacts generated in saline solution. (A) Bench-top recording settings. SPES was delivered every 2000 ms, with 60 trials recorded for each pulse shape. Each stimulation pulse shape was defined by five parameters. d1, dp, and d2 define the length of each pulse phase. a1/a2 define the amplitude of the negative/positive pulse phase, respectively. (B) We generated 110 different stimulation pulse shapes from combinations of d1 (33, 66, 100, 200, 300 *µ*s), d2 (33, 66, 100, 200, 300 *µ*s), dp (0, 66, 100, 200, 300 *µ*s), a1 (100 *µ*A) based on the commonly used configuration settings (Albert *et al* 2009). The summation of d1, dp, and d2 was restricted to not exceed 600 *µ*s, with a2 defined as $a2 = (d1*a1)/d2$. (C) Temporal dynamics of saline artifacts for all pulse shapes prior to (top) and after (bottom) applying MPARRM (averaged across trials, *n* = 60). (D)–(E) Effect of sampling rate (1–10 kHz) on the ability to accurately record the amplitude and shape of the SPES artifact. (F) Performance of MPARRM in removing SPES artifacts. For each pulse shape, we calculated the signal fluctuation (standard deviation) during baseline period (1000 ms to 200 ms preceding the stimulation onset) and artifact period (1 ms preceding and 1 ms following stimulation onset). We then calculated the signal fluctuation ratio between the artifact and baseline periods. A ratio equal to ∼1 means that the stimulation artifact has been completely removed. MPARRM effectively removed the artifact from the signal, irrespective of the sampling rate and pulse shape. Specifically, 1 kHz: 9.4 ± 8.2 to 0.91 ± 0.09; 2 kHz: 11.9 ± 7.9 to 0.92 ± 0.09; 2.5 kHz: 13.0 ± 8.0 to 0.93 ± 0.08; 5 kHz: 14.8 ± 11.8 to 0.92 ± 0.08; 7.5 kHz: 18.0 ± 11.5 to 0.95 ± 0.08; and 10 kHz: 19.3 ± 10.6 to 0.92 ± 0.06 (before/after MPARRM, mean ± s.d., *n* = 110).

Another fundamental issue is that the shape of stimulation artifacts is not identical across trials. This is because neural signals are usually recorded at a sampling rate that is too low (e.g. 1 kHz) to fully capture the shape of the individual artifacts in response to a 0.1–1 ms long stimulation pulse. Consequently, the shape of individual stimulation artifacts varies substantially across trials (figures 2(D) and (E), Sun and Hinrichs 2016). MPARRM addressed this issue by performing the stimulation artifact removal on a trial-by-trial basis. Specifically, MPARRM performed the analysis for each stimulation trial and decomposed the stimulation artifact from the general characteristics of that trial.

An artifact-affected signal typically consists of transient structures related to electrical stimulation (i.e. the stimulation artifact) and rhythmic/transient neural responses. To preserve rhythmic/transient neural responses, it is essential to extract the neural signals using techniques that can represent both rhythmic and transient components of that signal. Standard signal processing techniques such as IIR/FIR filters, short-time Fourier transform, or wavelet transform are all designed to preserve rhythmic components but fail to preserve sharp transient responses. MPARRM addressed this issue by applying a MP algorithm (Chandran KS *et al* 2016), which is a multiscale decomposition technique based on an over-complete dictionary, that has been demonstrated to capture both sharp stimulus-related transient activity and sustained rhythms in local field potentials (figure 1(A)).

Signals recorded during electrical stimulation are typically comprised of three major components (figure 1(B)): electrical line noise, sharp stimulation artifact, and neural activity that typically includes both rhythmic and transient electrophysiological signals. We aim to remove the stimulation artifact while preserving rhythmic/transient activity. We found that sharp Gaussian and Dirac (1 at t = 0; otherwise 0) atoms can represent sharp and transient signals; Gabor atoms provide a good compromise between frequency and time resolution; and Fourier (pure sinusoids) atoms can represent periodic signals such as line noise. Thus, our analysis combines sharp Gaussian, Dirac, Gabor, and Fourier atoms.

In detail, we developed a denoising method (i.e. MPARRM) capable of removing individual stimulation artifacts while preserving early spectral responses. The MPARRM denoising procedure is comprised of seven steps. For each step, the accompanied signal is labeled with a Roman numeral (I–VII, figure 1(C). In steps 1–2, we apply the MP algorithm to extract line noise and remove it from the signal. This approach overcomes several limitations of traditional notch filtering that often result in incomplete noise removal and introduce artifactual oscillations (i.e. ringing) around sharp stimulation artifacts (Ray *et al* 2008b). In steps 3–5, we apply the MP algorithm to extract the evoked potential and remove it from the signal to ensure that the evoked potential is not falsely identified as a stimulation artifact. This is because early negative potentials (N1) typically occur at 10–50 ms post-stimulus (Matsumoto *et al* 2004). The sharp transient morphology of these early potentials might be falsely identified as artifacts. In steps 6–7, we apply the MP algorithm to extract the stimulation artifact and remove it from the signal. The MATLAB code that implements MPARRM and sample data is available on GitHub (https://github.com/neurotechcenter/MPARRM_SPES).

• **Step 1:** decomposing the raw signal (I) using the MP algorithm to reconstruct the line noise (II) based on criterion $\#$1 (i.e. long atoms with a frequency above than 55 Hz).
• **Step 2:** removing the line noise from the raw signal (i.e. III = I–II).
• **Step 3:** interpolating the stimulation time window with a straight line from 2.5 ms preceding, to 2.5 ms following stimulation onset (IV).
• **Step 4:** decomposing the interpolated signal with MP algorithm to reconstruct the evoked potential (V) based on criterion $\#$2 (i.e. atoms with frequency below 70 Hz).
• **Step 5:** removing the evoked potential from signal-III (i.e. VI = III–V).
• **Step 6:** decomposing the residual signal (VI) with MP algorithm to reconstruct the stimulation artifact (VII) based on criterion $\#$3 (i.e. short atoms, centered ±5 ms around stimulation onset, represented by atom frequencies above 70 Hz or a Dirac atom).
• **Step 7:** removing the stimulation artifact from signal-III (i.e. VIII = III–VII). The green trace (VIII) represents the final denoised signal. The stimulation artifacts are removed while the electrophysiological neural signals (including the evoked potentials) are preserved.

### 2.2. Validating MPARRM on synthetic signals

To validate MPARRM, we performed a bench-top experiment in which we suspended electrodes in a saline solution to generate real-world stimulation artifacts. We then added the generated stimulation artifacts to a ground truth signal (i.e. human neural responses to auditory stimuli), applied MPARRM to the combined signal, and compared the resultant denoised signal with the ground truth signal. We expected effective denoising results with a strong correlation between the denoised and ground truth signals.

*Human stereoelectroencephalography (SEEG) signals recorded during receptive speech processing were used as ground truth signals.* The signals were recorded from nine human subjects (three males, six females, aged 22 to 46, supplementary figure 3(A), who underwent placement of SEEG electrodes (PMT, platinum/iridium contacts with 0.8 mm in diameter, contact length 2 mm, insulation length 1.5 mm) for intractable epilepsy treatment. The institutional review board at Washington University in St. Louis approved this study, and all subjects provided informed consent prior to participating in the study. SEEG signals were recorded using a Nihon Kohden JE-120 amplifier (Nihon Kohden, Tokyo, Japan) and the BCI2000 general-purpose brain-computer interface software (Schalk *et al* 2004). SEEG signals were amplified and digitized at 2 kHz. We utilized preoperative MRI imaging to produce three-dimensional brain models with Freesurfer, and localized implanted electrodes through co-registration of postoperative CT scans using SPM and subsequent processing in an intracranial electrode localization tool (i.e. Versatile Electrode Localization Framework, VERA, Adamek *et al* 2022). We used a receptive speech paradigm to evoke a cortical response. The stimuli consisted of 32 unique words presented to patients via over-ear headphones (12 Hz–23.5 kHz audio bandwidth, 20 dB isolation from environmental noise). The duration of each stimulus was 700 ms, and the inter-stimulus interval was 1000 ms. A total of 60 stimuli were presented.

*Stimulation artifacts generated in saline solution.* We generated isolated stimulation artifacts by suspending two standard clinical SEEG electrodes in a 0.9% saline solution (PMT, Chanhassen, MN). Similar to the clinical recordings, one SEEG electrode was used for stimulation and a second for recording. Both electrodes were placed in a saline bath which simulated the clinical environment to record stimulation artifacts (figure 2(A)). Stimulation was performed using a 16-channel headstage (M4016) controlled by the Intan Stim/Recording System (Intan Tech., USA). SPES was delivered through a middle contact of the first SEEG electrode using a monopolar configuration. The stimulation artifact was simultaneously recorded from a middle contact of a second SEEG electrode connected to a separate headstage. Stimulation was administered at a rate of 0.5 Hz with an inter-stimulus interval of 2000 ms. A total of 110 different biphasic pulse shapes were generated (figure 2(B), supplementary figure 2). Stimulus parameters were based on commonly used configuration settings (Albert *et al* 2009), and each pulse was analyzed across 60 trials. The resulting signal composed of stimulation artifacts was amplified and digitized at a sampling rate of 30 kHz. To generate a template of the saline SPES artifact for each pulse shape, we extracted the recording around each stimulus pulse (2000 ms preceding to 2000 ms following onset) and averaged the signal across all trials to reduce the background noise. We further reduced the sampling rate of the saline-based SPES artifact to 2 kHz (MATLAB $decimate()$) to match the sampling rate of human SEEG recordings.

**Figure 3.**
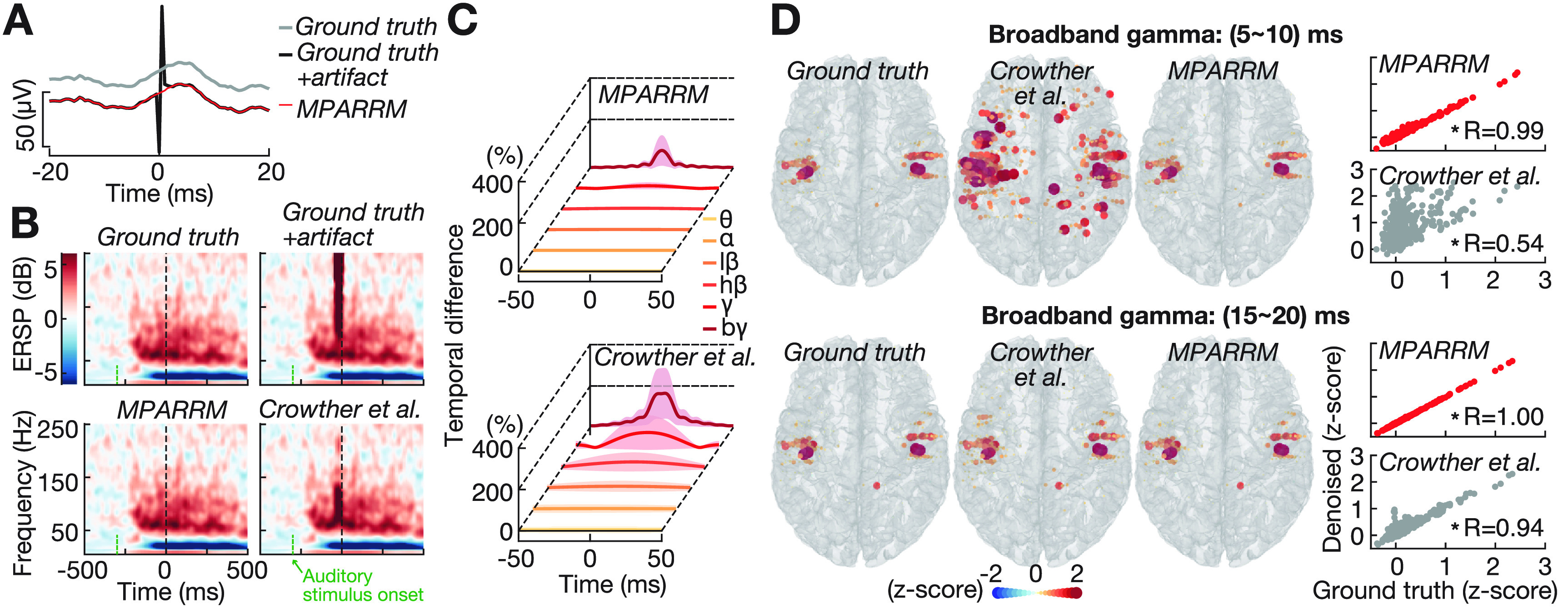
Validating MPARRM using synthetic signals with a representative pulse shape. (A) One representative single trial of ground truth signal (grey), ground truth signal added with saline stimulation artifact (black), and the denoised signal after applying MPARRM (red). We defined the ground truth signal as the SEEG signals recorded while performing a receptive speech paradigm. (B) Representative event-related spectral perturbation (ERSP) induced by an auditory stimulus for ground truth signal (top-left), ground truth signal added with saline stimulation artifact (time 0, top-right), the denoised signal after applying MPARRM (bottom-left), and the denoised signal after applying an interpolation-based stimulation artifact denoising method (bottom-right, Crowther *et al* 2019). (C) Temporal difference between the ground truth signal and the denoised signal within canonical frequency bands for MPARRM (top) and interpolation-based (bottom) denoising methods. (D) Left panel: topographic distribution of auditory-related broadband gamma response for ground truth signal (left) and the denoised signal for MPARRM (middle) and interpolation-based (right) denoising methods during two early-response periods. Right panel: correlation between the broadband gamma response of ground signal and denoised signal using MPARRM (upper, red) and interpolate (lower, grey) methods. Each dot indicates the averaged broadband gamma response during each response window of each electrode (* *p* $\lt$ ^0.001^, Pearson correlation).

*Validation of MPARRM.* We processed the human SEEG recordings by visual inspection and rejected those that exhibited artifactual activity. The signals were then high-pass filtered at 0.5 Hz to remove slow drifts. We re-referenced signals using a common average reference spatial filter (Liu *et al* 2015), extracted individual trials (1000 ms preceding to 700 ms following auditory stimulus onset), and randomly selected 50 signal channels from each subject (450 channels in total) to reduce the computational time. We added the saline SPES artifact around 300 ms following the auditory stimulus onset, where it showed a strong auditory-induced response (figure 3(B)), and then applied MPARRM to remove the stimulation artifact. We validated MPARRM results within the 0–30 ms-long period following saline stimulation onset for all canonical frequency bands, i.e. theta (*θ*, 4–7 Hz), alpha (*α*, 8–12 Hz), low beta ($l\beta$, 13–20 Hz), high beta ($h\beta$, 20–30 Hz), low gamma ($l\gamma$, 30–50 Hz), and broadband gamma ($b\gamma$, 70–170 Hz). Specifically, we filtered the ground truth signal and denoised signal (after MPARRM) from each electrode into each canonical frequency band, and calculated the absolute value of the Hilbert transform of the resulting signal. We then baseline-corrected and normalized the individual trials for each electrode by subtracting the average power during each trial’s baseline period (500 ms to 100 ms preceding the auditory stimulus onset) and dividing it through the standard deviation across all trials’ baseline periods. To maintain the alignment of the signal with the stimulation onset, we used forward-backward filters in our signal processing ($pop\_eegfiltnew()$ in EEGLAB, Delorme and Makeig 2004).

### 2.3. Testing MPARRM with basolateral amygdala stimulation

To further test MPARRM in general SPES studies, we applied our method to stimulation-artifact-affected neural signals recorded from the hippocampus while performing SPES on the ipsilateral basolateral amygdala. Because amygdala and hippocampus are anatomically and functionally connected (Inman *et al* 2018, Sawada *et al* 2022), we expected SPES to elicit early spectral responses in the hippocampus.

The signals were recorded from nine human subjects (seven males, two females, aged 24 to 66, supplementary figure 3(B) who underwent placement of SEEG electrodes for intractable epilepsy treatment. The institutional review board at Washington University in St. Louis approved this study, and all subjects provided informed consent to participate. We used the same recording setting as the receptive speech paradigm described above. During SEEG recording, 30–200 consecutive electrical pulses (biphasic, pulse width 200 *µ*s, current amplitude 6 mA, 0.5–1 Hz) were delivered to pairs of adjacent SEEG contacts located within the basolateral amygdala. We visually inspected and rejected those SEEG contacts that exhibited artifactual activity due to broken electrodes (i.e. contacts that did not record any physiological signals). We then re-referenced the remaining signals using the averaged signals across those channels that did not exhibit a strong CCEP response. Finally, we selected the SEEG signals within the ipsilateral hippocampus and applied MPARRM to remove their stimulation artifacts.

## 3. Results

### 3.1. Stimulation artifacts generated in saline solution

We performed a bench-top experiment to generate stimulation artifacts based on 110 different biphasic pulse shapes (figures 2(A) and (B)) that represent the most commonly used electrical stimulation settings (Albert *et al* 2009). We applied MPARRM directly to the artifact-affected signals from our bench-top experiment. Figure 2(C)–top shows the artifact-affected signals for different pulse settings. We expected MPARRM to substantially remove the artifact. Indeed, the results in figure 2(C)–bottom show that MPARRM reduced the average stimulation artifact amplitude from 34.1 *µ*V to 2.5 *µ*V. The results in figure 2(F) further confirm the ability of MPARRM to remove the stimulation artifact for different pulse shapes and sampling rates.

### 3.2. Example of early spatio-temporal response to SPES

Next, we verified the ability of MPARRM to remove the stimulation artifact while preserving the neural signal. For this first verification, we selected one representative stimulation setting (*d*1 = 100 *µ*s, *d*2 = 100 *µ*s, *dp* = 100 *µ*s, figure 2) to illustrate the MPARRM results. We defined the ground truth as the SEEG signals recorded while performing a receptive speech paradigm. We then added the artifacts generated in the saline stimulation experiment to the ground truth signal. Finally, we applied MPARRM to remove the stimulation artifact and obtain a denoised signal. Figure 3(A) shows one representative trial of ground truth signal (grey), added signal (black), and denoised signal after applying MPARRM (red). Time 0 indicates the onset of the SPES artifact. Figure 3(B) shows a strong event-related spectral perturbation (ERSP) increase around time 0 in the added signal, which is related to the stimulation artifact. This strong ERSP vanished after applying MPARRM. In contrast, the conventional interpolation denoising method (Crowther *et al* 2019) failed to remove this artifact. To evaluate the effectiveness of MPARRM in removing the stimulation artifact, we calculated the difference in spectral amplitude between the ground truth signal and the denoised signal. Specifically, we calculated the absolute value of the temporal difference (subtracting the denoised signal from the ground truth signal) within each frequency band, then divided the difference value by the averaged amplitude of the ground truth signal. Figure 3(C) shows the difference in spectral amplitude across all channels and all trials (mean ± s.d., *n* = 27 000). Compared to conventional interpolation, MPARRM-based denoising reduced the difference between ground truth and denoised signal within 0–20 ms post-stimulation onset in each frequency band (*θ*: 4.8% to 0.56%; *α*: 7.5% to 0.90%; $l\beta$: 15% to 1.9%; $h\beta$: 32% to 4.4%; $l\gamma$: 66% to 11%; $b\gamma$: 93% to 33%). The interpolation denoising method showed a larger deviation from ground truth than MPARRM, especially for broadband gamma (93% vs. 33%). Furthermore, we found that MPARRM yielded a topographic distribution of auditory-related broadband gamma in the early response window (5–10 ms after the stimulation artifact onset) that was almost perfectly aligned with that of the ground truth (*R* = 0.99, figure 3(D)–top). This is in marked contrast to the topographic distribution yielded by conventional interpolation, which exhibited a strong deviation from ground truth (*R* = 0.54). For the later response window (15–20 ms post-stimulation onset), MPARRM yielded perfect results with *R* = 1.00 (figure 3(D)–bottom).

### 3.3. Early responses to SPES after applying MPARRM

Next, we were interested in systematically quantifying the ability of MPARRM to preserve the early response within all canonical frequency bands. For this purpose, we compared the denoised signals (after applying MPARRM or interpolation method) with the ground truth signals. To quantify the performance of MPARRM to preserve the early response, we divided this time window into six 5 ms-long time bins, for which we then extracted the power signals of each trial. In our evaluation, we calculated the corresponding correlation, sensitivity, and specificity metrics. We performed the analysis for each frequency band and stimulation setting separately. We were especially interested in the early broadband gamma response, which has been shown to be tightly correlated with multi-unit spiking activity (Nir *et al* 2007, Ray *et al* 2008a, Manning *et al* 2009, Miller *et al* 2014).

**Figure 4.**
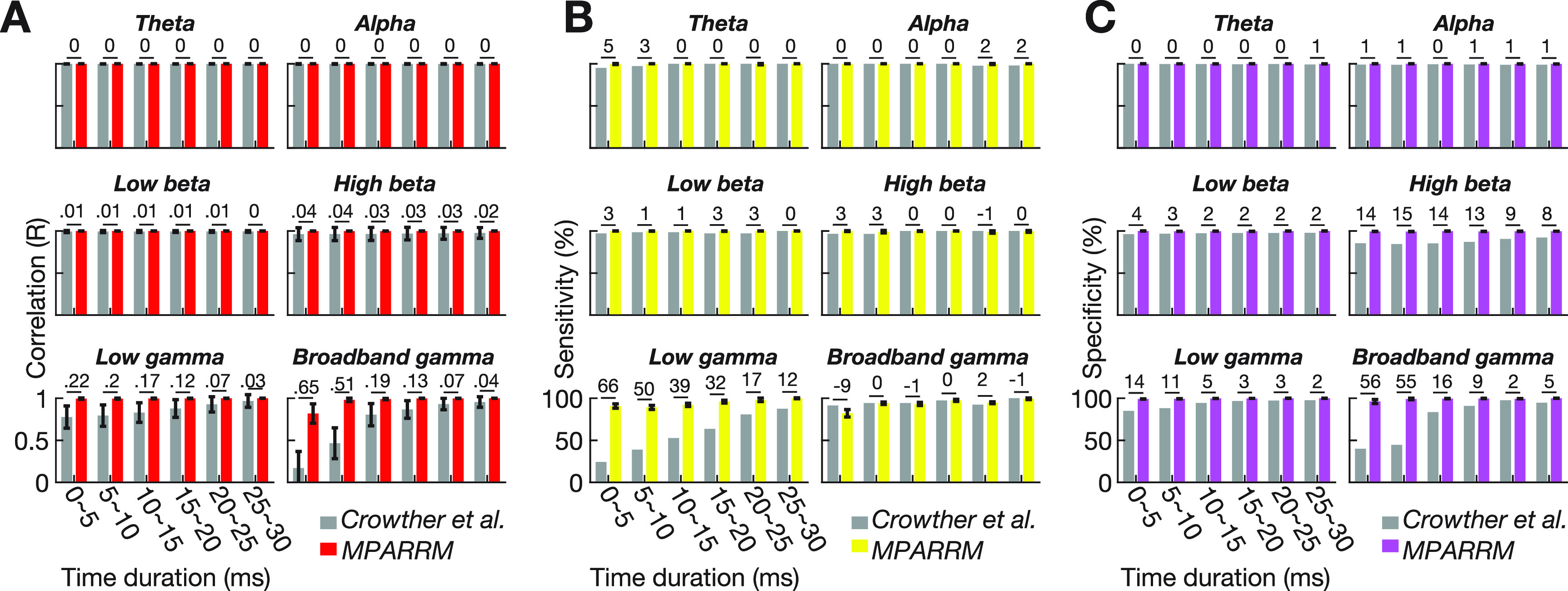
Validating MPARRM with synthetic signals. (A) Correlation between the denoised signal and ground truth signal within canonical frequency bands using MPARRM (red) and interpolation-based (grey) denoising methods. (B) Sensitivity for the denoised signal after applying MPARRM (yellow) and interpolation-based (grey) denoising methods when compared to the ground truth signal. (C) Specificity for the denoised signal after applying MPARRM (purple) and interpolation-based (grey) denoising methods when compared to the ground truth signal. The interpolation-based denoising method is described in Crowther *et al* (2019). The value above each bar shows the absolute difference between MPARRM and interpolation-based denoising methods.

To determine the correlation metric, we calculated the Pearson correlation *R* between band power (averaged across each time-point within each bin) of the denoised signal and the ground truth signal (mean ± s.d.; *n* = 49 500 for MPARRM across all pulse shapes and channels; *n* = 450 for interpolation denoising method across all channels; figure 4(A). The results of this analysis demonstrate the excellent performance of MPARRM in preserving the very early responses (5–10 ms) with an averaged *R* value equal to 0.98 ± 0.02 for broadband gamma and a *R* value close to 1 for the lower canonical frequency bands. In contrast, the conventional interpolation yielded a markedly lower *R* value for broadband gamma (*R* = 0.47 ± 0.18). Of note, for the later response window (25–30 ms), MPARRM and interpolation became equally efficient in removing the stimulation artifact.

To determine the sensitivity and specificity metrics, we added the SPES artifacts, recorded in our bench-top experiment, to the neural signals at 300 ms post-auditory stimulus onset (figure 3(B)). This ensured a temporal overlap between the expected strong power modulation induced by the auditory stimulus and the onset of the SPES, thus allowing us to quantify the ability of MPARRM to preserve the early response to SPES in the presence of physiological responses. To do this, we first identified those channels that contained a significant neural response to the auditory stimuli. Specifically, we performed a one-sample *t*-test of the bin mean band power value for each time step of each electrode (the power signals were *z*-scored to the baseline, see Methods). We expected channels with *p* < 0.01 (two-tailed) to exhibit a significant neural response to auditory stimuli. Next, we calculated the sensitivity (figure 4(B)) and specificity (figure 4(C)) of the denoised signal to faithfully reveal the responses to the auditory stimuli within the ground truth signal for each canonical frequency band (supplementary figure 4). Specifically, we calculated the average sensitivity and specificity along each time step across all pulse shapes for MPARRM (mean ± s.d., *n* = 110). The results of this analysis show that MPARRM performs well in preserving very early neural responses (5–10 ms), with an average sensitivity and specificity of 94% ± 1.47% and 99% ± 1.01% for broadband gamma, respectively. Sensitivity and specificity reached almost 100% for the lower frequency bands. In marked contrast, the results based on conventional interpolation exhibit a much lower specificity in preserving the very early broadband gamma response (45%). For later responses, MPARRM and interpolation denoising perform equally well.

### 3.4. Early spatio-temporal responses to human basolateral amygdala stimulation

**Figure 5.**
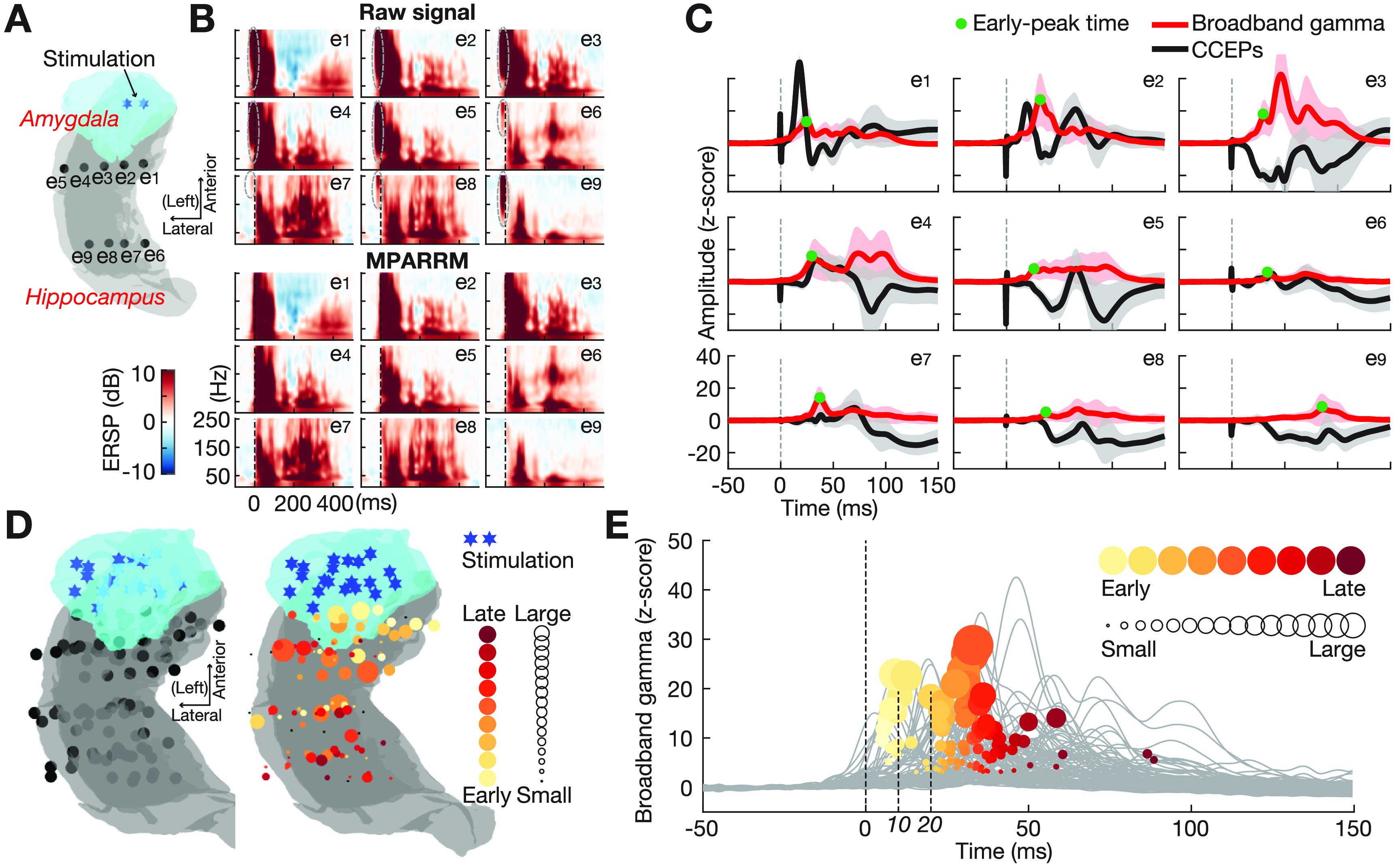
Validating MPARRM on SPES-responses recorded from human amygdala-hippocampus circuit. (A) Stimulation location (blue asterisk) within basolateral amygdala (blue area) and recording sites (black dots) within ipsilateral hippocampus (grey area) for one representative subject. (B) Upper panel: event-related spectral perturbation (ERSP) of the raw signal. Grey-dashed circles highlight confounding artifact-related power increases around stimulation onset. Lower panel: ERSP after applying MPARRM. Confounding artifact-related power increases around the stimulation onset are not present after stimulation artifact removal by MPARRM. (C) CCEPs (mean ± s.d., black line, without stimulation artifact removal) and extracted broadband gamma (mean ± s.d., red line) after applying MPARRM. Green dots indicate the early-peak time of the broadband gamma responses. (D) Left panel depicts stimulation and recording sites across all subjects. For visualization purposes, we projected right hemispheric recording sites onto the left hemisphere (supplementary figure 3(B). Right panel shows the early-peak time of the broadband gamma responses recorded from locations along the anterior-posterior axis of the hippocampus. The yellow/red color represents the early-peak time ranging from early (5 ms, bright yellow) to late (100 ms, dark red). The circle size indicates the strength of broadband gamma responses. (E) Averaged broadband gamma response of each recording site. We found that 16% of the recording sites (17/104) show an early peak time within 20 ms of stimulation delivery.

We were interested in further validating the ability of MPARRM in preserving the neural response to SPES. For this purpose, we applied MPARRM to stimulation-artifact-affected neural signals recorded from the human hippocampus while delivering SPES to the ipsilateral basolateral amygdala. We expected that SPES delivered to the basolateral amygdala would elicit responses throughout the ipsilateral hippocampus. We first verified that MPARRM sufficiently removed the stimulation artifact. Specifically, we expected no significant neural activity around stimulation onset time after applying MPARRM. Indeed, the results for one representative subject in figures 5(A)–(C) show that MPARRM sufficiently removed the artifact-related power centered around the stimulation onset (figure 5(B)).

Next, we determined the temporal propagation of SPES-induced neural activity throughout the hippocampus. We expected to find a temporal progression of the SPES-induced neural activity in the broadband gamma band along the anterior-posterior axis of the hippocampus. To test this hypothesis, we detected the early-peak time of the SPES-induced broadband gamma activity at each recording site by choosing the first peak of the averaged broadband gamma that exceeded three times the standard deviation calculated during pre-stimulation baseline (figure 5(E)). The results of our analysis exhibit a temporal progression along the anterior-posterior axis of the hippocampus (figure 5(D)).

## 4. Discussion

We developed the novel MPARRM technique to remove the stimulation artifact induced by SPES. MPARRM is a model decomposition method with three outstanding characteristics. First, it performs the stimulation artifact removal on a trial-by-trial basis, without assuming a constant morphological shape across trials. Second, it can be applied to variable stimulation parameters (e.g. pulse shapes) and does not need to be adjusted for specific pulse shapes, which makes MPARRM a robust analysis tool. Lastly and most importantly, MPARRM can recover neural signals after stimulation, which provides a unique opportunity to probe the early response of SPES.

At its core, MPARRM is based on the MP algorithm. MP, as originally proposed by Mallat and Zhang in 1993 (Mallat and Zhang 1993, Chandran KS *et al* 2016), is an iterative decomposition technique that approximates a time-domain signal using a linear combination of waveforms called atoms. MP-based algorithms have been used to separate spikes from oscillatory or broadband activity (Ray *et al* 2008b, Jmail *et al* 2011, Ray and Maunsell 2011, Ray *et al* 2013 Salelkar *et al* 2018), to estimate the duration of gamma activity (Chandran KS *et al* 2018), to detect epileptic activity (Franaszczuk *et al* 1998, Goelz *et al* 2000, Z-Flores *et al* 2016, Khlif *et al* 2022), and to extract single-trial evoked potentials (Sieluzycki *et al* 2009, Jörn *et al* 2011). Specifically, MPARRM extracts line noise, evoked potentials, and stimulation artifacts by sequentially applying the MP algorithm. Previous studies demonstrated the ability of the MP algorithm to extract line noise (Ray *et al* 2008b), single-trial evoked potentials (Sieluzycki *et al* 2009, Jörn *et al* 2011), and signals with sharp shapes (e.g. spike and epileptic activity (Ray and Maunsell 2011, Khlif *et al* 2022)). In our study, we further assessed the advantages of using the MP algorithm over other traditional signal decomposition methods (including short-time Fourier transform (STFT), multitaper method (MTM), and wavelet transform (WT)) in isolating the stimulation artifact in the time and frequency domain. For this purpose, we applied these methods to neural signals without (supplementary figure 6(B) and with (supplementary figure 6(C) stimulation artifacts. The results of this comparison unequivocally show that the MP algorithm is particularly well-suited to isolate the temporal and spectral characteristics of neural signals from those of stimulation artifacts. Together, the results of these studies support the notion that MPARRM accurately removes the stimulation artifact without affecting the physiological components of the electrophysiological signal.

Early responses of SPES could potentially provide vital insight into local neural circuitry. Prior studies have shown early response in CCEPs (Toth *et al* 2020, Kudela and Anderson 2021) and neural firing patterns (Douglas and Martin 1991, Alarcón *et al* 2012). However, our understanding of the spectral responses during the early response period is still very limited, mainly because the stimulation artifact largely contaminates the signals nearby due to the effects of signal filtering (de Cheveigné and Nelken 2019). The spectral responses carry a multitude of important information, e.g. the power spectral changes of the broadband gamma signals could represent the average firing rate of neurons located directly underneath the recording electrodes (Manning *et al* 2009, Whittingstall and Logothetis 2009, Miller *et al* 2014). In our study, we have demonstrated that MPARRM can effectively remove the stimulation artifact while preserving the early response of broadband gamma (within 5 ms to 10 ms following stimulation onset). Specifically, we showed that broadband gamma activity after applying MPARRM is highly correlated with the ground truth signal (*R* = 0.98, Pearson correlation, figure 4(A). Our control analysis showed that traditional interpolation-based methods could not preserve this early response (*R* = 0.47, Pearson correlation, figure 4(A).

Another potential application of MPARRM is to explore the early response generated by deep brain stimulation (DBS) for Parkinson’s disease (PD). DBS electrodes for PD treatment are implanted in the subthalamic nucleus (STN) or globus pallidus internus (GPi), and generate evoked potentials in both local (e.g. DBS local evoked potentials, DLEPs) and remote (e.g. cortical evoked potentials, cEPs) regions (Schmidt *et al* 2020, Dale *et al* 2022). DLEPs are recorded directly at or near the stimulation site and generally have two components: a short-latency evoked component (peak latency of about 0.31 ms) and a long-latency component (starting at 4.5 ms post-stimulation). cEPs are recorded from the motor cortex and generally exhibit three components in response to low-frequency stimulation within the STN ($\unicode{x2A7D}$20 Hz), i.e. short- (R1, 1–3 ms), intermediate- (R2, 5–15 ms), and long- (R3, 18–25 ms) latency responses. DBS-evoked potentials arise from a complex integration of antidromic and orthodromic conduction pathways and may provide biomarkers for improving DBS outcomes and function (Dale *et al* 2022). For example, evoked potentials may have utility as control signals for DBS programming or adaptive DBS. As discussed above, the early response to DBS can occur within less than 25 ms of stimulation onset, making it difficult to extract the corresponding early spectral response due to the presence of the stimulation artifact. MPARRM can overcome this issue by preserving the early spectral response to electrical stimulation. Thus, studies applying MPARRM to DBS response could potentially reveal the spatio-temporal dynamics of DLEPs and cEPs, and lead to new DBS-related biomarkers for improving DBS outcomes and function.

MPARRM removes the artifact on a trial-by-trial basis. Thus, MPARRM does not depend on information from other trials or channels. Because of this ability, MPARRM can accommodate artifact shapes that vary across different channels, time, and even sampling rates (supplementary figure 5). Because of the greedy fashion of MPARRM, it can remove artifacts resulting from a wide range of pulse shapes (supplementary figure 2). Furthermore, the ability to remove stimulation artifacts on a single-trial basis makes the MPARRM technique well-suited for real-time applications, such as closed-loop adaptive neuromodulation (Guidetti *et al* 2021). However, the approximation of signals using the MP algorithm requires an iterative approach (Pati *et al* 1993) that generally requires more computational time than other denoising methods (e.g. interpolation). In our study, we partially address this issue by testing the limits in reducing the number of necessary iterations. Specifically, we found that reducing the number of iterations from 50 to 10 still yielded satisfactory results in removing the stimulation artifacts (supplementary figure 7).

Additionally, there are several issues and limitations to MPARRM. (1) The underlying MP algorithm can be configured to use a wide range of dictionaries. We thus need to select the dictionary that best suits the signal to be analyzed. In our study, we chose dictionaries that involve Gabor, Gaussian, and Dirac atoms, which have been verified to be particularly well-suited to represent the various temporal and spectral characteristics of local field potential signals (Ray and Maunsell 2011, Chandran KS *et al* 2016, 2018). (2) MP chooses atoms in a greedy fashion in an attempt to maximize the energy of the modeled signal at each iteration. If it selects an ‘inappropriate’ atom (e.g. one that does not represent a biological phenomenon), subsequent iterations will yield atoms that will attempt to correct for this mistake and, therefore, yield more inappropriate functions (Chen *et al* 2001). (3) Our analysis assumes that the stimulation artifact is contained within a 10 ms-long window (i.e. 5 ms preceding to 5 ms following stimulation onset), that the activity within this window is a combination of the neural signal and a stimulation artifact, and that the spectro-temporal characteristics of the stimulation artifact do not overlap with those of the neural signal. However, specific circumstances may violate these assumptions. For example, elongated refractory periods may extend the artifact window, amplifier saturation may completely destroy the signal during this period, and the neural response to electrical stimulation within specific local circuits could overlap in its spectro-temporal characteristics with those of the stimulation artifact. Under these circumstances, MPARRM needs to be adapted to account for these challenging conditions. For example, the artifact window could be extended, and more specific criteria for separating the neural signal from the stimulation artifact could be introduced.

## 5. Conclusions

MPARRM presents a robust solution for the removal of SPES-related artifacts. It can faithfully remove the stimulation artifact without confounding the electrophysiological signal components. Specifically, it allows extracting the spectral responses within the early stimulation-response period, which could have a great impact on both basic neuroscientific studies and neurological therapies.

## Data Availability

We are fully sharing the MATLAB code that implements MPARRM along with the necessary sample data to reproduce and inspect the methodological results shown in the manuscript (https://github.com/neurotechcenter/MPARRM_SPES). In addition, full datasets will be provided to interested researchers upon reasonable request to the corresponding authors. These full dataset includes data from our clinical studies, which we can only share in de-identified form with other institutions upon request. The data that support the findings of this study are available upon reasonable request from the authors.
